# Periodic genome sequences facilitate packaging in a single-stranded DNA virus

**DOI:** 10.1128/jvi.00701-26

**Published:** 2026-07-01

**Authors:** Elizabeth T. Ogunbunmi, Megan Mokriski, April D. Burch, Bentley A. Fane

**Affiliations:** 1 The BIO5 Institute, University of Arizona 124486, Tucson, Arizona, USA; 2 Department of Molecular and Cellular Biology, University of Arizona 8041, Tucson, Arizona, USA; 3 Science Department, Berkshire School 108990 , Sheffield, Massachusetts, USA; Michigan State University, East Lansing, Michigan, USA

**Keywords:** DNA packaging, Microviridae, phiX174

## Abstract

Icosahedral viruses organize and compact their genomes within volumetrically constrained capsids. Double-stranded (ds) DNA viral genomes form ordered spool-like structures when packaged into preformed capsids. By contrast, single-stranded (ss) RNA viral coat proteins recognize genomic sequences or structures, nucleating assembly around folded genomes. A similar mechanism may occur in some ssDNA systems; however, in parvo- and microviruses, the ss genome is concurrently synthesized and packaged into a preformed shell. In the øX174 X-ray virion structure, only ~12% of the genome is ordered. Consequently, the mechanism by which the genome is accommodated within the constrained capsid remains obscure. Sequence motifs within the øX174 genome produce a periodic segmentation pattern consistent with T = 1 icosahedral symmetry. To determine the function of these motifs, a subset within the first five packaged segments was altered. No regulatory elements or encoded amino acids were changed. The resulting mutant, øX^DO5^, displayed a phenotype consistent with those of previously characterized packaging mutants. To further understand this phenomenon, the DNA replication-packaging pathway was characterized in cells co-infected with wild-type and øX^DO5^. Each genome could both be separately tracked and distinguished throughout the pathway. Early, pre-packaging øX^DO5^ genome replication was comparable to the wild-type. However, øX^DO5^ genomes were severely diminished within virions, suggesting a defect in the transition from packaging intermediates to mature virions.

**IMPORTANCE:**

Concurrent genome biosynthesis and packaging are specific to some families of single-stranded (ss) DNA icosahedral viruses. This evolutionary strategy combines elements found in both dsDNA and ssRNA systems. Like dsDNA viruses, the genome is packaged into a preformed capsid. Like ssRNA viruses, there are numerous capsid-genome associations. However, in microviruses, such as øX174, these interactions do not facilitate capsid assembly around the genome. They occur after the ss genome enters the preformed procapsid. Sequence motifs within the øX174 genome produce a periodic segmentation pattern consistent with T = 1 icosahedral symmetry. The data provided herein demonstrate that altering these periodic motifs can lead to packaging defects, suggesting an additional level of selective pressure acting on ssDNA genomes.

## INTRODUCTION

There are two general strategies for packaging single-stranded (ss) genomes into icosahedral capsids. (i) ssRNA genomes assume highly compact tertiary folds that closely approximate capsid volume (1–3). Capsid proteins bind specific genomic sequences or structures that nucleate assembly around the RNA (4–7), a process referred to as packaging signal-mediated assembly (8). (ii) While a similar mechanism may occur in some ssDNA viruses, micro- and parvoviruses package their genomes into a preformed shell. Therefore, the periodic sequences investigated herein do not function as capsid assembly signals. Irrespective of the packaging strategy, nucleic acid concentrations can approach ~500 mg/mL (9). To achieve these high concentrations, encapsidated nucleic acids must be compacted and organized.

### øX174 DNA synthesis and packaging

**Fig 1:**
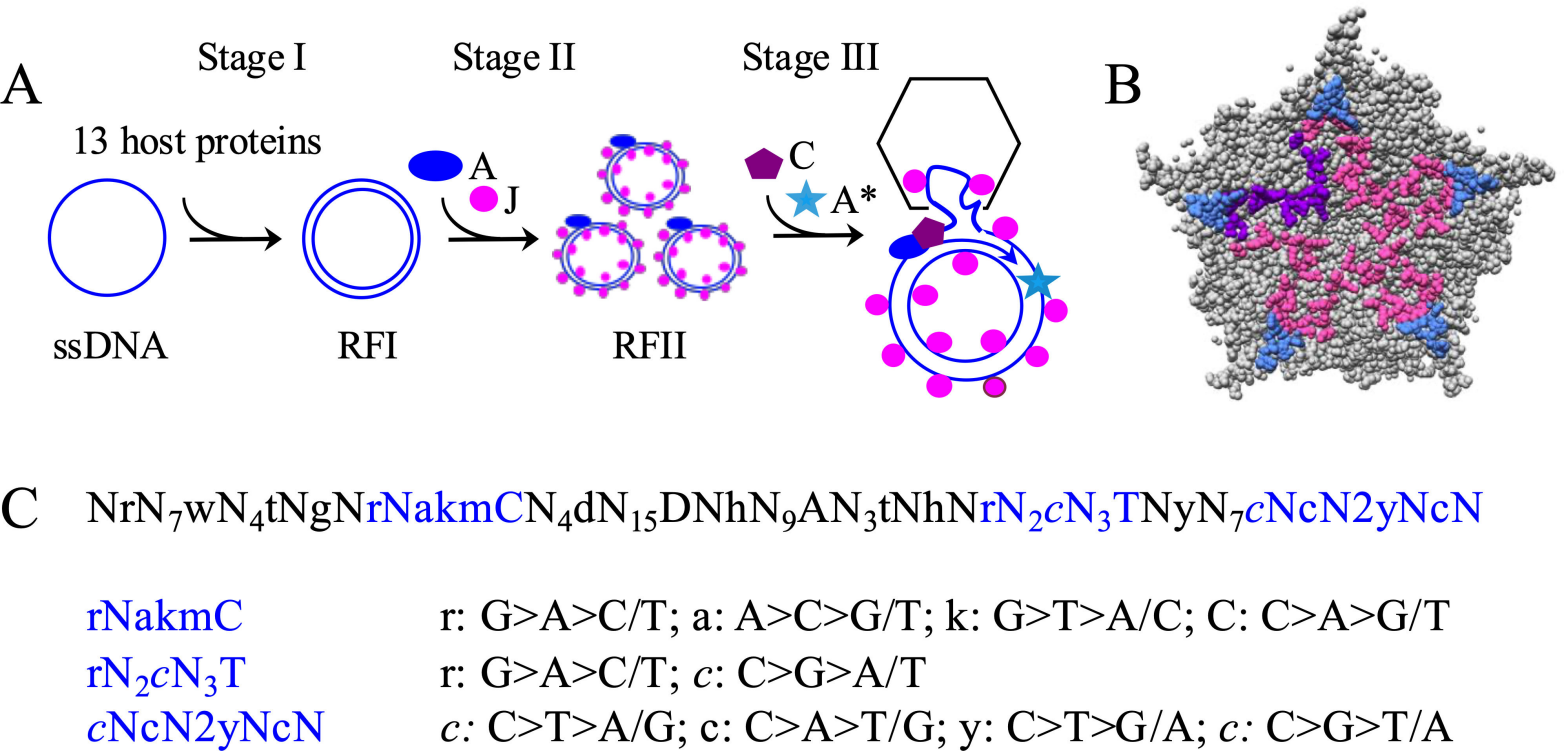
øX174 DNA packaging and the periodic sequence. (**A**) The three stages of øX174 replication. See the text for details. (**B**) Organization of the J protein and ordered DNA genome around five-fold axes of symmetry (PDB 2BPA). The coat proteins are depicted in gray. One J protein is highlighted in purple; the other four are depicted in magenta. Blue represents the ordered DNA backbone within the DNA-binding pocket. (**C**) The consensus periodic sequence (top). The nucleotides most found in each position are given below the sequence. The blue text depicts the most statistically significant computational predictions.

øX174 DNA replication occurs in three distinct stages (Fig. 1A). In Stage I, 13 host-cell proteins convert the infecting ssDNA genome to a double-stranded molecule referred to as replicative form (RF) DNA (10). After viral protein synthesis, Stage II rolling circle replication is initiated by protein A, which covalently binds to the origin of replication (11–14). Amplification of the double-stranded RF DNA is inhibited by protein C, marking the beginning of Stage III DNA synthesis (15, 16). Each RF molecule is coated with ~120 copies of the DNA-binding protein J (17). In Stage III, genome synthesis and packaging are tightly coupled concurrent processes (18, 19). Both *in vitro* and *in vivo*, an ssDNA genome is not synthesized unless there are procapsids into which it can be inserted (19, 20). The parental strand is displaced into the procapsid as the daughter strand is synthesized (Fig. 1A). In the X-ray virion structure, protein J appears to guide the genome between DNA-binding pockets around the five-fold axes of symmetry (Fig. 1B). In these pockets, the genome’s phosphate backbone is ordered, constituting ~12% of the DNA (21). After one round of rolling circle replication, protein A ligates the two ends of the packaged linear parental strand to complete the reaction (12).

When two processes are tightly coupled, a degree of synchronization is likely required. In the øX174 system, daughter strand synthesis cannot outpace parental strand packaging. If this occurred, unpackaged ssDNA would accumulate outside the procapsid. There, it could form secondary structures, which may not pass through the 30-Å-wide packaging pore, or non-productively interact with the replicative-form dsDNA template. The A* protein (A star) appears to maintain this synchronicity. It binds target sequences in the dsDNA template and hinders the host-cell *rep* helicase (22). Elevated protein A* levels rescue some packaging mutants and extend the lower temperature range of productive packaging (23–25).

The origin of replication and A* target sites are defined *cis*-acting elements involved in ssDNA replication and packaging. Spatially, they influence these processes outside the procapsid. The results of *in vitro* packaging studies suggest that there may be a third *cis*-acting element that affects genome organization inside the capsid. If the origin of replication is present, bacteriophage øX174 can package unit-length foreign DNA. However, packaging is less efficient when compared to the wild-type genome. Many reactions do not go to completion, and ~90% of the packaged particles lack infectivity (26). Near wild-type efficiency can be achieved by reducing the size of the foreign DNA template to ~91% unit length (26).

The above observations suggest the øX174 genome has specific motifs optimizing the packaging reaction, which may be difficult to detect, obscured by the genetically compressed nature of the genome (27). Reading frames overlap with each other and with many *cis*-acting elements: promoters, ribosome-binding sites, transcription terminators, A* target sites, and the origin of DNA replication. Thus, novel and more complex computational analyses may be required to detect subtle motifs dispersed throughout the genome. Chechetkin and Lobzin found periodic motifs in several bacteriophage and plant virus genomes (28). In the øX174 genome, the spacing of these motifs is consistent with the T = 1 icosahedral symmetry of the major capsid protein, as well as the stoichiometry of DNA-binding protein J. In this study, a genetic and biochemical analysis was conducted to determine the possible impact of these periodic motifs on packaging or other processes.

## RESULTS AND DISCUSSION

### Alterations to the periodic sequences resulted in a cold-sensitive phenotype

The possible impact of the periodic sequences on packaging and other processes was characterized by deoptimizing a subset of motifs (Fig. 1C). Deoptimization was performed by replacing wild-type nucleotides with less commonly found ones in those positions. For example, in the “rNAkmC” motif, the “r” position is most commonly a G, followed by an A, and then a C or T. If the G could be changed without altering the protein sequence or any *cis*-acting elements, it was changed to the least common nucleotide at that site. In this example, it would be a T.

Packaging is directional beginning after the origin of replication. The genome was altered by oligonucleotide-mediated mutagenesis. Deoptimization began after the origin, which partially overlaps with the first periodic segment. Mutations were added sequentially, approximately seven at a time, until the virus no longer displayed a wild-type phenotype. Again, changes were made only if genetic elements or protein-coding sequences remained unaltered. The resulting mutant, øX^DO5^, contained 39 nucleotide changes (Fig. S1) from positions 4331 to 4715. This region approximately encompasses the first five periodic segments of 60 to be packaged. It displayed a cold-sensitive (cs) phenotype (Table 1), which was rescued by the exogenous expression of a cloned A* gene, a characteristic often associated with packaging mutants (23, 29).

**TABLE 1:** Plating efficiency and specific infectivity of øXDO5

Strain	Plating efficiency^*a*^			Strain	Specific infectivity^*c*^	
	37°C	24°C^*b*^				
		−A*	+A*		37°C	24°C
øX174	1.0	0.6	1.0	am(E)	3.7 × 10¹²	3.1 × 10¹¹
øXD05	1.0	10⁻³	0.2	øX^D05^	1.8 × 10¹²	1.2 × 10¹¹

^*a*^ Plating efficiency: 37°C titer/24°C titer. For plating efficiency, WT øX174 was used.

^*b*^ 24°C titers were determined with and without the exogenous expression of a cloned A* gene.

^*c*^ PFU/*A*_260_. Specific infectivity was calculated with the am(E) phage used in sedimentation analyses. Temperature refers to the temperature at which the particles were synthesized.

### Infectivity of purified øX^DO5^ and am(E)W4 virions

The results of past analyses defined two general classes of packaging mutations (24, 29, 30). Some mutations cause packaging complexes to dissociate or move into the insoluble fraction. These mutants are rescued by increased A* gene expression. The second class of mutations allows packaging to go to completion, but the particles lack infectivity, a phenotype that is not rescued by elevated A* gene expression. To further distinguish between these two possibilities, progeny generated at 24° and 37°C in lysis-resistant cells were purified by rate zonal sedimentation in 5%–30% sucrose gradients. After fractionation into approximately 40 125 µL fractions, the virion-containing fractions were detected by *A*_260_ and plating assays. Specific infectivity was defined as PFU/*A*_260_. No dramatic differences were observed (Table 1). For these experiments, øX174 am(E)W4 was used as the “WT” control. The am(E)W4 mutations confer an amber mutation in the fourth codon of gene E, which encodes the lysis protein. No other function has been attributed to the E protein, and all infections were conducted in lysis-resistant cells. Moreover, the amber phenotype allowed infection products to be differentially titered in the co-infections described below.

### Rationale for conducting the øX^DO5^ and am(E)W4 co-infections

øX^DO5^ exhibits cold sensitivity and protein A*-mediated rescue, indicative of packaging mutants. However, past studies have focused on *trans*-acting proteins (23, 24), as opposed to the DNA packaging substrate. Unlike a mutant protein, which can be complemented in *trans*, the øX^DO5^ packaging defect should be *cis*-acting in co-infections with am(E)W4. Moreover, the co-infection controls for unforeseen and/or undetectable effects on gene expression by deoptimization. The presence of the wild-type genomes should mitigate those effects, yet the hypothesized *cis*-acting øX^DO5^ packaging defect should remain. This was tested in two ways. First, relative yields of each phenotype were determined by differential titering. However, the relative titers only reflect the ratio of the end products: fully packaged, plaque-forming particles. Therefore, the individual genomes were tracked throughout the entire DNA replication pathway (described below).

### Respective fates of øX^DO5^ and am(E)W4 genomes in co-infected cells

**Fig 2:**
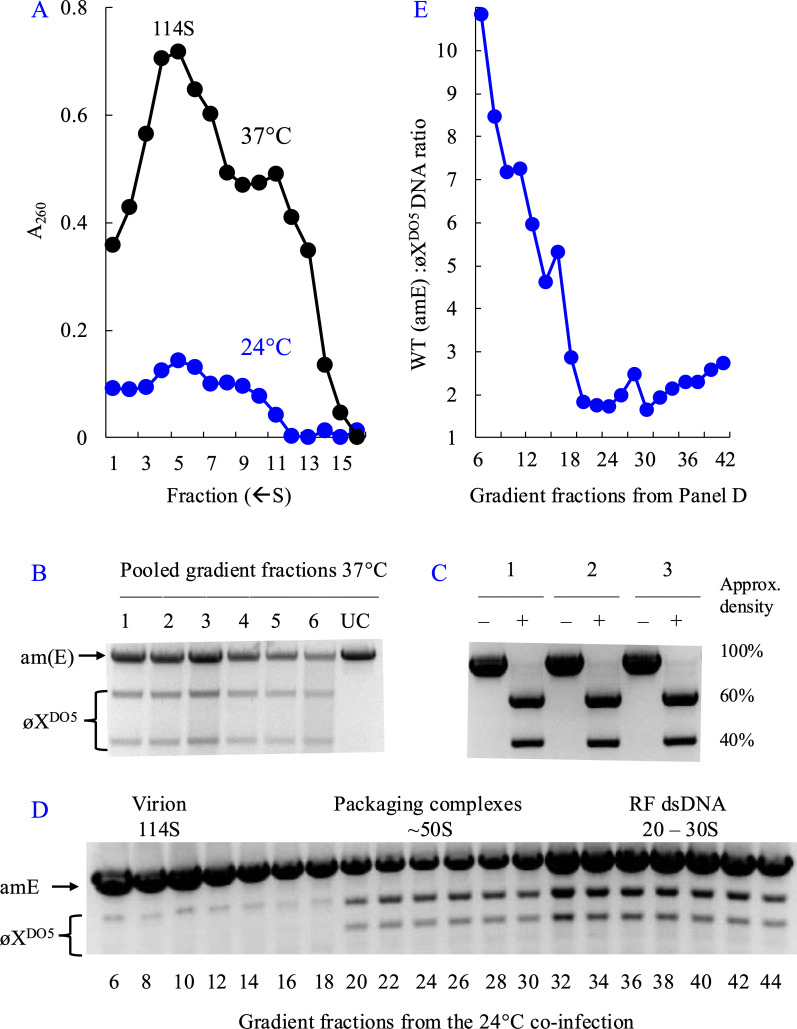
*In vivo* characterization of am(E)W4 and øX^DO5^ co-infection products. (**A**) Sedimentation profiles of 37°C (black) and 24°C (blue) co-infections. Mature virions sediment at 114S. (**B**) PCR and restriction digests from am(E) and øX^DO5^ genomes in virions and replicating intermediates from the 37°C co-infection. Sucrose gradient fractions (~45) were pooled, and the DNA in the fraction was PCR-amplified and digested with Nhe I and run on an agarose gel. Pool 1 corresponds to the virion peak, pool 2 to partially packaged or empty procapsids, and pools 3 to 6 cover packaging complexes and RF DNA. (**C**) Normalization of restriction band intensities. Each PCR (1, 2, and 3) was divided into two equal aliquots. Only one was digested with Nhe I. The digested and undigested samples were loaded side by side on an agarose gel. An image of the gel was digitized. The relative band intensities of the two digested bands are given in the figure. (**D**) Even fractions of the 24°C co-infection sucrose gradient were PCR-amplified and digested with Nhe I. The location of virions and replicating intermediates is given within the figure. (**E**) Graphic representation of densitometry normalization of WT am(E) and øX^DO5^ DNA bands in panel D.

Lysis-resistant host cells were co-infected with øX174 am(E)W4 and øX^DO5^, each at a multiplicity of infection (MOI) of 3.0, at 37°C and 24°C. Lysates were prepared as described in Materials and Methods. Infectious virions were separated from early DNA replication intermediates by rate zonal sedimentation. Virions (114S particles) are the product of Stage III DNA replication, concurrent ssDNA synthesis, and packaging (Fig. 1A), whereas Stage I and Stage II replication produce replicative form dsDNA templates, which sediment between 20S and 30S. After centrifugation, the 5 mL gradients were divided into approximately 40 125-µL fractions. The resulting 37°C and 24°C sedimentation profiles are depicted in Fig. 2A. At both temperatures, a clear virion peak was detected in the earlier fractions.

The progeny in the virion peaks were differentially titered. To obtain a total titer, am(E)W4 and øX^DO5^ progeny, samples were plated on the informational suppressing cell line BAF8 (*supF*), whereas the wild-type C122 (*sup°*) host was used to obtain an exclusive øX^DO5^ titer. The respective BAF8 and C122 titers of the 37°C progeny were 3.5 × 10^12^ PFU/mL and 2.0 × 10^12^ PFU/mL, corresponding to an am(E)W4:øX^DO5^ ratio of ~3:4. Thus, the two genomes were packaged in approximately equal amounts at the permissive temperature. By contrast, the respective BAF8 and C122 titers of the 24°C infection were 3.7 × 10^10^ PFU/mL and 6.4 × 10^9^ PFU/mL, which corresponds to a 6:1 am(E)W4:øX^DO5^ ratio. Thus, at lower temperatures, fewer øX^DO5^ genomes appear to flow entirely through the DNA replication pathway.

Titers, however, do not track the individual genomes throughout the DNA replication pathway. As described in the Introduction (Fig. 1A), Stage I and II replication occur independently of the concurrent genome biosynthesis and packaging of Stage III replication. The øX^DO5^ mutations were constructed in a background containing a unique and silent Nhe I restriction site, which is located 1,100 bases away from the last substitution in the øX^DO5^ sequence. DNA replication intermediates are easily separated from virions. Replicative form (RF) DNA, the products of Stage I and II replication, sediments between 20S and 30S, whereas packaging complexes and virions respectively sediment at 50S and 114S.

Figure 2B displays data generated from the 37°C co-infection. Fractions were pooled based on the well-characterized sedimentation rates of intermediates. Pools 1–2 contained virions (fractions 1–15 in Fig. 2A), pools 3–4 contained packaging intermediates, and pools 5–6 contained RF DNA. DNA was PCR-amplified and digested with Nhe I, producing three bands. The largest band represents øX174 am(E) DNA (no Nhe I site), and the two smaller bands represent øX^DO5^ DNA (Nhe I site). To calculate a genome ratio between the phages, the intensities of the smaller bands were quantified relative to the larger undigested band (Fig. 2C). To calculate the constant that approximates this relationship, PCR products were generated from the øX^DO5^ single infection (Fig. 2C). Each PCR was divided into two equal aliquots. Before electrophoresis, one aliquot was digested with Nhe I. Using ImageJ analysis software, the bands were assigned quantitative values. The respective pixel intensities of the larger and smaller Nhe I restriction fragments were ~ 60% and ~40% of the undigested band. Thus, an approximate øX174 am(E) to øX^DO5^ DNA ratio in a co-infection is given by the following formula:


$$\frac{\emptyset Xam(E)}{\emptyset XDO5}=\frac{\left(0.6)(\text{intensity undigested band}\right)}{\text{intensity large }Nhe\text{ I restriction fragment}}$$

Applying this formula to the DNA gel in Fig. 2B resulted in an am(E)W4:øX^DO5^ DNA ratio of approximately 1:1 throughout the entire gradient.

A similar analysis was conducted with the fractions from the 24°C co-infection. However, fractions were not pooled; instead, even-numbered fractions encompassing the entire gradient were analyzed (Fig. 2D). There did not appear to be a dramatic difference between am(E)W4 and øX^DO5^ DNA in the RF DNA-containing fractions. Thus, the øX^DO5^ DNA mutations did not appear to affect Stage I and II DNA replication. However, the ratio began to skew toward am(E)W4 DNA in intermediate fractions, which contain packaging complexes, and even more dramatically in the virion fractions. An approximate quantitative ratio was calculated as described above and graphed (Fig. 2D). In agreement with the gel, the graph shows that the am(E)W4:øX^DO5^ DNA ratio increased to ~10:1 in the virion fractions. These data suggest that øX^DO5^ packaging complexes were less stable or less soluble than wild-type packaging complexes at 24°C. Thus, the defect conferred by the øX^DO5^ substitutions was *cis*-acting.

While the experiments herein provide the “proof of concept” that the periodic motifs affect packaging, they do not define the associated mechanism or their evolution. Therefore, further discussion is brief and highly speculative. In a direct mechanism, these motifs may orchestrate specific genome-capsid interactions forming pinning contacts on the inner surface of the capsid. In an indirect mechanism, the periodic sequences may affect the genome’s persistence length, which measures the flexibility of a polymer. Persistence length increases at lower temperatures (31), resulting in a less flexible packaging substrate, which is consistent with øX^DO5^’s cold-sensitive phenotype and the general cold-sensitivity of microvirus packaging (24). An evolutionary model should account for three features of the øX174 system: (i) the periodic motifs overlap extensively with all coding sequences; (ii) microvirus ssDNA biosynthesis and packaging are tightly coupled; and (iii) albeit inefficiently, microviruses can package foreign DNA (26). Thus, the earliest evolutionary pressure was likely on components most integral for function: capsid and packaging complex morphogenesis. Afterward, redundancy in the genetic code allowed the system to evolve subtle components, while minimizing disruptions to the most integral ones. This likely explains the degeneracy of the periodic sequences (Fig. 1C).

## MATERIALS AND METHODS

### Phage plating, media, buffers, stock preparation, bacterial strains, and plasmids

The plating protocol, media, buffers, stock preparation, and the wild-type *Escherichia coli* C strain C122 and BAF8 (*supF*) strain have been previously described (32). The RY7211 cell line used to generate the infected cell lysate contains a mutation in the *mraY* gene, conferring resistance to viral E protein-mediated lysis (33). The cloned øX174 A* gene is under arabinose induction, was induced by supplementing media with 0.2% arabinose, and repressed by supplementing media with 0.2% glucose (29).

### Site-directed mutagenesis to generate øX^DO5^

The mutagenesis strategy is described in Results. Briefly, the 39 mutations were added sequentially, approximately seven changes at a time. In each round, the entire genome was PCR-amplified with Q5 DNA polymerase (NEB) using abutting primers that introduce the mutations. The PCR products’ 5′ hydroxyl termini were phosphorylated and ends ligated using T4 polynucleotide kinase and ligase (NEB).

### Infected cell extracts, extracts for gradient preparation, and protein electrophoresis

The protocols for rate zonal sedimentation and protein electrophoresis have been described in detail previously (34–36). To generate infected cell extracts for sucrose gradients, 100 mL of lysis-resistant cells was infected at an MOI of 3.0 (single infections) or 6.0 (co-infections), followed by incubation for 3 h at 37°C and 6.5 h at 24°C. Infected cells were concentrated, resuspended in 3.0 mL of sucrose gradient buffer (100 mM NaCl, 5.0 mM EDTA, 6.4 mM Na_2_HPO_4_, and 3.3 mM KH_2_PO_4_ [pH 7.00]), and then lysed by overnight incubation with lysozyme (2.0 mg/mL). After cellular debris was removed by centrifugation (10 min at 20,000 × *g*), the resulting supernatant was concentrated to 200 µL in a Nanosep centrifugal filter column (100-kDa cutoff). The extract was then loaded atop a 5.0-mL, 5 to 30% sucrose gradient (wt/vol) and spun at 192,000 × *g* for 1 h. Gradients were divided into approximately 40 125-µL fractions, and assembled particles were detected by UV spectroscopy (*A*_260_ and *A*_280_). To examine whole-cell lysates before centrifugation, 1.0 mL of infected cells was concentrated (3 min at 20,000 × *g*) in a benchtop microfuge. Pellets were resuspended in 100 µL SGB (see above). Aliquots were mixed with SDS-PAGE loading buffer and boiled before SDS-PAGE.

### Determination of the genotype ratios in DNA replicating intermediates and virions

To distinguish between am(E)W4 and øX^DO5^ genomes, the DNA in gradient fractions was PCR-amplified using GoTaq (Promega) following the manufacturer’s instructions. After amplification, Nhe I (NEB) was added directly to the PCRs and incubated per the manufacturer’s instructions. Products were separated by agarose gel electrophoresis, and an image of the gel was digitized. The band intensity was analyzed using ImageJ analysis software. Ratios were determined as described in the Results.

## Data Availability

All relevant data are within the paper and its supplemental material.

## SUPPLEMENTAL MATERIAL

Fig. S1 jvi.00701-26-s0001.pdf Sequence alignment of øX^DO5^ with wild-type øX174.

ASM does not own the copyrights to Supplemental Material that may be linked to, or accessed through, an article. The authors have granted ASM a non-exclusive, world-wide license to publish the Supplemental Material files. Please contact the corresponding author directly for reuse.
