# Supplementary material for: Periodic genome sequences facilitate packaging in a single-stranded DNA virus
Source: J Virol. 2026 Jul 1;100(7):e00701-26. doi: 10.1128/jvi.00701-26 (PMC13386984; doi:10.1128/jvi.00701-26)
Supplement: Fig. S1 — Sequence alignment of øXDO5 with wild-type øX174. [file jvi.00701-26-s0001.pdf]

```
øXDO5    GATATTAATAACACTATAGACCACCGTCCCGAAGGTGATGAAAAATGGTTTCTTGAAAACGAAAAAACTGTCACTCAGTT
         :::::::::::::::::::::::::: :::::::: :: :::::::::::: : :: ::::: :: :: :: :: :::::
WT       GATATTAATAACACTATAGACCACCGCCCCGAAGGGGACGAAAAATGGTTTTTAGAGAACGAGAAGACGGTTACGCAGTT
               4310      4320      4330      4340      4350      4360      4370      4380

øXDO5    TTGCCGCAAGCTGGCTGCTGAACGTCCTCTCAAAGACATTAGAGATGAATACAATTACCCCAAAAAGAAAGGTATTAAGG
         :::::::::::::::::::::::: ::::: :: :: ::: : ::::: :: ::::::::::::::::::::::::::::
WT       TTGCCGCAAGCTGGCTGCTGAACGCCCTCTTAAGGATATTCGCGATGAGTATAATTACCCCAAAAAGAAAGGTATTAAGG
               4390      4400      4410      4420      4430      4440      4450      4460

øXDO5    ATGAGTGTTCAAGATTGCTGGAGGCCTCCACTATGAAATCGCGCCGTGGGTTTGCTATTCAGCGTTTGATGAACGCAATG
         :::::::::::::::::::::::::::::::::::::::::::  : :: ::::::::::::::::::::::: ::::::
WT       ATGAGTGTTCAAGATTGCTGGAGGCCTCCACTATGAAATCGCGTAGAGGCTTTGCTATTCAGCGTTTGATGAATGCAATG
               4470      4480      4490      4500      4510      4520      4530      4540

øXDO5    CGACAAGCTCACGCTGACGGTTGGTTTATCGTTTTTGACACTCTCACGTTGGCTGATGATAGATTAGAAGCGTTCTATGA
         ::::: ::::: ::::: :::::::::::::::::::::::::::::::::::::: ::  ::::::: ::::: :::::
WT       CGACAGGCTCATGCTGATGGTTGGTTTATCGTTTTTGACACTCTCACGTTGGCTGACGACCGATTAGAGGCGTTTTATGA
               4550      4560      4570      4580      4590      4600      4610      4620

øXDO5    TAACCCCAACGCTTTGCGTGACTATTTTCGTGATATTGGTCGTATGGTTCTTGCTGCGGAGGGTCGCAAGGCTAACGATT
         ::: ::::: ::::::::::::::::::::::::::::::::::::::::::::::: ::::::::::::::::: ::::
WT       TAATCCCAATGCTTTGCGTGACTATTTTCGTGATATTGGTCGTATGGTTCTTGCTGCCGAGGGTCGCAAGGCTAATGATT
               4630      4640      4650      4660      4670      4680      4690      4700

øXDO5    CACACGCCGATTGCTATCAG
         :::::::::: :::::::::
WT       CACACGCCGACTGCTATCAG
               4710      4720
```

Supplemental Figure 1. Sequence alignment of the øXDO5 with wild-type øX174. The numbers refer to the nucleotide position within the øX174 genome.
